# Effects of Different Dietary Energy Levels on Development, Quality of Carcass and Meat, and Fatty Acid Profile in Male Lambs

**DOI:** 10.3390/ani13182870

**Published:** 2023-09-09

**Authors:** Dan Zhang, Chao Yuan, Tingting Guo, Jianbin Liu, Zengkui Lu

**Affiliations:** 1 Key Laboratory of Animal Genetics and Breeding on the Tibetan Plateau, Ministry of Agriculture and Rural Affairs, Lanzhou Institute of Husbandry and Pharmaceutical Sciences, Chinese Academy of Agricultural Sciences, Lanzhou 730050, China; 2 Sheep Breeding Engineering Technology Research Center of Chinese Academy of Agricultural Sciences, Lanzhou 730050, China

**Keywords:** alpine merino, energy, growth performance, meat quality

## Abstract

**Simple Summary:**

Alpine Merino sheep are a new breed of sheep adapted to the cold and arid ecological zone 2400–4070 m above sea level; the breed has important economic significance for high-altitude areas. This study evaluated the effects of different dietary energy levels (9.7 MJ/kg, 10.1 MJ/kg, 10.5 MJ/kg) on the growth and meat quality of weaned Alpine Merino lambs. The results showed that compared with the low-energy diet (9.7 MJ/kg), the high-energy (10.5 MJ/kg) diet promoted the growth and development of lambs, increased the live weight and carcass weight of lambs before slaughter, significantly reduced the yellowness and redness scores of lamb muscles, and increased the meat’s content of monounsaturated fatty acids.

**Abstract:**

This experiment was conducted to study the effects of dietary energy level on the growth performance and meat quality of weaned Alpine Merino lambs. The study ran for a total of 104 days (20-day pretrial, 84-day trial). From three groups of test lambs, we randomly selected ten lambs per group to determine slaughter performance, meat quality characteristics, and organ indexes. The slaughter performances of the lambs improved as the dietary energy level increased. The live weight before the slaughter of the lambs was significantly higher in the high group than in the low and medium groups. The carcass weight was significantly higher in the high group than in the low group. Dietary energy level had little effect on the organ weight of lambs. Meat quality differed among the three dietary energy levels. The muscle yellowness and redness scores decreased significantly as the energy levels increased. The C18:0, C21:0, C20:1, C18:2n6c, and C20:2 contents in the muscle were significantly higher in the high group than in the medium and low groups. The C18:3n6 content in the muscle was significantly higher in the low group than in the medium group. The C20:5n3 content in the longissimus dorsi was significantly higher in the high group than in the medium and low groups. The monounsaturated and unsaturated fatty acid contents in the muscle were significantly higher in the high group than in the low group. A dietary energy level of 10.5 MJ/kg is suitable for fattening weaned male Alpine Merino lambs.

## 1. Introduction

China is rich in sheep resources and has a long history of raising sheep. The Peiligang site in Henan Province indicates that the history of sheep raising in China dates back 8000 years [1]. In feudal society, ancient populations regarded sheep with respect and considered lamb to be the best meat. As economic levels improve, lamb is gradually becoming a common meat. Compared with beef and chicken, lamb is richer in nutrients and contains more lysine, arginine, histidine, methionine, and riboflavin [2]. Alpine Merino sheep are the newest breed of wool and meat producers that are adapted to cold and dry alpine ecological zones at altitudes of 2400–4070 m. Alpine Merino sheep are mainly distributed in the cold and dry pastoral areas of Qilian Mountain at the eastern foot of the Qinghai Tibetan Plateau, where it is dry and cold in spring and winter and warm and humid in summer and autumn [3]. As Alpine Merino breeding increases, the sheep’s wool yield and quality of meat production performance are gaining attention.

Energy is an important limiting factor for nutrition [4]. One study showed that the metabolic energy intake of 70 kg Alpine Merino rams gaining 100 g per day was 16.64 MJ/d [5]. Another study showed that at the grass-eating stage, the metabolic energy intake of 70 kg Alpine Merino rams was 9.6 MJ/d [6]. Energy deficiency slows lamb growth and reduces lamb quality [7,8]. Increasing dietary energy levels can increase daily weight gain and promote lamb growth and development [8,9]. Fatty acid contents in meat are determined by interactions between diet and rumen microbes, limiting the level of metabolizable energy that can reduce intramuscular fat deposition [10]. Fatty acids are the main factors affecting meat flavor and determine the taste and nutritional level of the meat [11,12]. Meat quality is directly related to muscle fiber type [13]. Skeletal muscle consists of approximately 90% muscle fibers and 10% connective tissue and fat. Muscle fibers are classified into four types, (I, IIa, IIx, and IIb) and are associated with myosin heavy chain (MyHC) [14]. When energy intake is restricted, type I muscle fiber increases and type IIB fiber decreases [15], indicating that muscle fiber type and MyHC expression levels are correlated with dietary energy level [16], and muscle fatty acid content was correlated with muscle fiber type [17]. Thus, dietary energy level affects muscle fiber type and intramuscular fatty acids.

Alpine Merino sheep are mainly raised via grazing but face problems with insufficient foraging and poor grazing quality in the winter, in addition to small breeding scale, low fattening efficiency, and potential to damage the local environment. Supplementary feeding or fattening in different areas is necessary for farmers. Currently, no reports exist on the appropriate dietary energy levels for weaned Alpine Merino lambs. Therefore, we evaluated three energy levels in weaned male Alpine Merino lambs to study how these energy levels affect growth and meat quality characteristics and explore the nutritional requirements appropriate for lamb growth and fattening. We further explored the effects of half-barn feeding and breeding in stables to provide a technical reference.

## 2. Materials and Methods

### 2.1. Animals, Diets, and Experimental Procedures

Forty-five 4-month-old weaned male lambs weighing approximately 24 kg each were selected and randomly divided into three groups of fifteen per group. Single-column feeding was used to feed each group a supplemented diet with one of three energy levels. The feeding period was 104 days, including a 20 d pretrial period and an 84 d formal trial period. The formal trial period was divided into three 28 d periods. During the 20 d pretrial period, the lambs were gradually introduced and adapted to a complete pellet diet rather than foraging. During the formal trial period, the lambs were fed a strictly pelleted diet at 8:00 a.m. and 17:00 p.m. daily. The feeding amount was adjusted each morning so that the remaining feed amount accounted for ~15% of the feeding amount. During the feeding period, it was ensured that there was free and clean drinking water for the sheep, the sink was cleaned regularly, the sheep house was cleaned every day, and the environment was kept clean. During the feeding period, the test sheep were vaccinated in accordance with epidemic prevention requirements. The animal study was reviewed and approved by the Institutional Animal Care and Use Committee of Lanzhou Institute of Husbandry and Pharmaceutical Science of Chinese Academy of Agricultural Sciences (Approval No. NKMYD201805; Approval Date: 18 October 2018).

The diet was corn–soybean meal type, designed according to the American NRC (1985) meat sheep feeding standard (dry matter design) [18]. The high-energy group was supplemented with an energy level of 10.5 MJ/kg; the medium-energy group was supplemented with 10.1 MJ/kg, and the low-energy group was supplemented with 9.7 MJ/kg. Table 1 shows the dietary components.

### 2.2. Sample Collection

Ten Alpine Merino lambs were randomly selected from each experimental group, and their live weights were recorded. These 30 lambs were exsanguinated via the jugular vein in compliance with veterinary policy rules after fasting for 24 h. After slaughter and exsanguination, the skin, viscera (except the kidneys), and surrounding fat were removed, and then the head, wrist joint of the forelimb, tarsal bone of the hind limb, and the part below the toe joint were removed. Thirty minutes later, the whole body was weighed and recorded as the hot carcass weight. Next, the bilateral longissimus dorsi muscles were completely separated, and samples of the digestive tract, testicles, and other organs of all lambs were collected from all experimental lambs onsite post slaughter and transported back to the laboratory at 4 °C to determine the meat quality.

### 2.3. Average Daily Gain

At the end of each trial period, all experimental lambs were weighed before the morning feeding, and the data were recorded to two decimal places to calculate the lambs’ average daily gain. The average daily gain was calculated as the total weight gain of the lambs during the test period/total days of the test.

### 2.4. Carcass Characteristics

The lambs were prepared, and the hot carcass weight was recorded as described in Section 2.2. The dressing percentage was the ratio of the carcass weight of the test lambs with the kidneys pre-served to the live weight before slaughter (LWBS), expressed as a percentage: dressing percentage = carcass weight (kg)/live weight before slaughter (kg) × 100.

Outlines of the longissimus dorsi of the test lambs were drawn on sulfuric acid paper, the maximum length and width were marked, and the cross-sectional area was calculated to determine the EMA (eye muscle area): EMA (cm^2^) = eye muscle height (cm) × eye muscle width (cm) [19].

### 2.5. Meat Quality

A portable pH meter (HI 99163; Hanna Instruments, Woonsocket, RI, USA) was calibrated with two buffers (pH 7.01 and 4.01) and then used to measure the pH of the muscle samples. A metal rod was used to make a hole in the middle of the muscle being cut, and an acidity-measuring electrode was inserted so that the meat was attached to the spherical end of the electrode. The muscle pH was measured 45 min after slaughter and again after storage at 20 °C for 24 h [20].

Fresh meat samples were stored for 45 min, then chilled in a refrigerator at 4 °C for 24 h. Meat color was measured after 45 min and 24 h. The lens of the colorimeter (OPTO-STAR, MATTHAUS, Germany) was placed vertically on the longest cross-section at the last rib of the back for measurement. Each sample was measured 5–8 times, and the muscle lightness (L*), redness (a*), and yellowness (b*) values were recorded [20].

To determine the water loss rate, the lumbar longissimus dorsi muscle was cut, and a cylindrical eye muscle sample of 1.0 cm thickness and 2.5 cm diameter was cut perpendicular to the muscle fiber direction with a 2.5 cm diameter circular sampler. Next, 18 layers of qualitative medium-speed filter paper were placed on each side, and the sample was pressed to 35 kg for 5 min. The weight of the meat samples was measured before and after pressurization (with the filter paper absorbing surface moisture) using a balance with a precision of 0.001 g. The water loss rate was calculated as follows: water loss rate = (weight before meat sample pressing—weight after meat sample pressing)/weight before meat sample pressing ×100 [20].

To determine the drip loss, the longissimus dorsi muscle was cut to 5 cm × 3 cm × 2 cm. After hanging in a refrigerator at 0–4 °C for 24 h, the weights of the meat samples before and after hanging were measured using a balance with a precision of 0.001 g. The drip loss was calculated as (weight before meat sample hanging—weight after meat sample hanging)/weight before meat sample hanging × 100 [20].

To determine the cooked meat percentage, ~100 g of the middle of one side of the longissimus dorsi muscle was used, and the fat attached to the muscle membrane was removed. The sample was steamed in an aluminum steamer with boiling water on an electric stove at 1000 W for 30 min. After cooling at room temperature for 15 min, the surface moisture was dried with filter paper. The percentage was calculated as the weight before cooking/weight after cooking ×100 [20].

To determine the shear strength, the eye muscle was refrigerated at 4 °C for 72 h, and the fascia and surface fat were removed and placed in an 80 °C water bath and heated to a core temperature of 70 °C. The sample was removed and hung in a cool dry place at room temperature, then stored at 4 °C overnight. Ten center meat samples (approximately 1.5 cm × 1.0 cm × 1.0 cm) were cut along the muscle fiber direction with a 1 cm diameter sampler, and then a tender shear force meter (Shanghai Baosheng Industrial Development Co., Ltd., Shanghai, China, TA. XTC) was used to measure the muscle shear force [20].

Next, 2 g of the eye muscle was removed after extracting the fat using a hydrolysis-ether solution. C11:0 was used as the internal standard. The column was an HP-88 (100 m × 0.25 mm × 0.2 μm) (Agilent Technologies, Santa Clara, CA, USA), with a column flow rate of 1 mL/min, 270 °C rear inlet temperature, 1 μL injection volume, and flow rate of 24.00 mL/min. The carrier gas was high-purity nitrogen filtered by a fully automatic air generator (Beijing Huilong), the front detector was FID, the temperature was 260 °C, and the gas flow rate was 30.00 mL/min. The initial column box temperature was 100 °C, held for 13 min, then heated to 180 °C at 10 °C/min for 6 min, then to 200 °C at 1 °C/min for 20 min, and then to 230 °C at 4 °C/min for 10.5 min, with a total running time of 85 min [21].

The meat sample was chopped, and 0.3 g minced meat and 15 mL of 6 N HCl (GR grade) (Sinopharm, Shanghai, China) were added to a sealed hydrolysis tube, which was placed in a hydrolysis furnace at 110 °C ± 1 °C for 22 h. The sample was filtered through filter paper after fixing the volumetric setting, and then the filtrate was concentrated in a rotating vacuum evaporator (Shanghai Ya Rong RE-52AA, Shanghai, China) at 55 °C until the solvent was completely removed. Next, 2.0 mL sodium citrate buffer (pH 2.2; Biochrom, Ltd., Cambridge, UK) was added to the dried test tube and dissolved. After shaking and mixing, the absorbed solution was passed through a 0.22 μm filter membrane and transferred to the instrument injection bottle for sample determination. Samples were analyzed using a Biochrom 30+ automatic amino acid analyzer (Biochrom Ltd., Cambridge, UK), with the following conditions: column type and sulfonic acid type: cationic resin; detection wavelengths: 570 and 440 nm (proline); separation column temperature: 57 °C; buffer flow rate: 0.35 mL/min; ninhydrin reagent flow rate: 0.35 mL/min; and injection volume: 1 μL [22].

### 2.6. Statistical Analysis

Data were analyzed using MS Excel for simple statistics, SPSS 23.0 for one-way ANOVA, and Duncan’s test for multiple comparisons, and significant differences between groups were analyzed.

## 3. Results

### 3.1. Body Weight and Daily Weight Gain

On day 104, the lamb body weights were significantly higher in the high group than in the low group (*p* < 0.05). The three energy levels did not significantly affect the daily gain. The growth rate of the medium group decreased, then increased in the third trial phase, which was similar to that of the low group and higher than that of the high group. The growth rate of the low group showed a slow downward trend, with the most weight being gained in the third trial stage (Table 2).

### 3.2. Slaughter Performance

The LWBS was significantly higher in the high group than in the medium and low groups, and the carcass weights were significantly higher in the high and medium groups than in the low group (*p* < 0.05). The dressing percentage was highest in the medium group and significantly differed from that of the low group (*p* < 0.05) (Table 3).

### 3.3. Organ Indexes

Organ development did not significantly differ among the three groups (*p* > 0.05). The testicular weight was higher in the medium group and lower in the high group. The small intestinal length increased as the energy level increased. The large intestines were longest in the high group and shortest in the medium group (Table 4).

### 3.4. Meat Physical Quality

The a* at 45 min was significantly lower in the high and medium groups than in the low group (*p* < 0.05). The muscle a* and b* at 24 h were significantly lower in the high and medium groups than in the low group (*p* < 0.05). The 45 min muscle pH was highest in the low group, and the 24 h muscle pH was highest in the high group. The water loss rate was higher in the high and low groups than in the medium group, and the shear force and drip loss were lower in the high and low groups than in the medium group. EMA did not significantly differ among the three groups (Table 5).

### 3.5. Intramuscular Fatty Acid Profiles

The unsaturated fatty acid content in the muscle was significantly higher in the high group than in the other two groups (*p* < 0.05). The C18:0, C15:1, C21:0, C21:1 and C20:2 contents in the muscle were significantly higher in the low group than in the other two groups (*p* < 0.05). C20:5n3 was significantly higher in the high group than in the other two groups (*p* < 0.05). The monounsaturated fatty acids were significantly lower in the low group than in the high group (*p* < 0.05). The saturated/unsaturated fatty acid ratio was significantly higher in the low group than in the medium and high groups (*p* < 0.05) (Table 6).

### 3.6. Amino Acid Contents in the Muscle

The total amino acid (TAA) contents did not significantly differ among the three groups (*p* > 0.05), but the Asp, Leu, Glu, and Lys contents were higher in the muscle, with the Glu content being the highest. The essential amino acid (EAA)/TAA ratio was >45% and was higher in the medium and low groups than in the high group, but not significantly (*p* > 0.05). The EAA/nonessential amino acid (NEAA) ratio was >80% in all groups and was lowest in the high group, but with no significant differences among the three groups (*p* > 0.05). (Table 7)

## 4. Discussion

Body weight is an important index for measuring the growth of fattening lambs. Sileshi et al. reported that low energy levels restricted the growth of experimental sheep and the absorption and use of dietary proteins [23]. Therefore, energy is an important condition affecting lamb growth and development. Studies have shown that the body weight and daily gain of lambs increased as the dietary energy level increased [4,24,25]. We found that the body weights of the lambs increased significantly as the dietary energy level increased, but the three energy levels did not significantly affect the daily gain. The body weight gap first increased, then decreased, because as the live weight of the fattening lambs increased, the body weight gradually approached its peak owing to the decreased energy-related metabolic proteins [26]. Among the three dietary energy levels, the high-energy diet was the most suitable for fattening lambs.

Slaughter performance is among the most important indicators and can easily and effectively indicate the economic return of fattening lambs. Carcass weight and dressing percentage are the main indicators for determining slaughter performance. Studies have shown that feed energy levels affect slaughter performance, and when energy levels in-crease, animals gain more weight, thus increasing the carcass weights and dressing percentage [27,28]. The nutrition of grazing sheep is limited by the forage abundance; when pasture-fed experimental sheep received extra supplements at different energy levels, the pre-slaughter weight, hot carcass weight, cold carcass weight, and dressing percentage increased as the feed energy level increased [29]. In this study, both the pre-slaughter and carcass weights increased as the energy level increased. Interestingly, our results show that the medium group had the highest dressing percentage, which was significantly higher than that of the low group. As lambs approach their adult weights, the bone, viscera, and fat weights increase [30], and the subcutaneous fat thickness increases with the feed energy level [31]. These factors may have resulted in the higher dressing percentage of the medium group. The EMA is an indicator of slaughter performance. Increasing the energy intake of lambs increases the EMA [32]. Studies have shown a positive correlation between the EMA of Merino sheep and carcass anatomical traits [33]. In our study, the EMA was higher in the medium group than in the other two groups, which was one reason for the higher dressing percentage of the medium group.

As the body matures, the internal organs also mature. During maturation, the physiological functions of the internal organs improve, and their volume and weight increase. The rumen is an important digestive organ in ruminants, and it develops faster than the reticulum, omasum, and abomasum in lambs’ early development. The intestinal tract is another important organ for digestion and metabolism; it is the final place of digestion and absorption and closely interacts with intestinal antigens and diverse bacterial communities, which is important for health [34]. A study showed that low maternal energy intake greatly affected the fetal muscles, bones, and organs and that the organ weights of the fetal liver, lungs, spleen, kidneys, heart, and large intestines were significantly lower in the low group than in the high group [35]. In fetal sheep, energy restriction negatively affects the muscles and reproductive organs, and the effects on the reproductive organs persist into adulthood [32]. In this study, the effects of the three energy levels on the internal organs did not significantly differ, but the spleen and liver weights were lower in the low group than in the medium and high groups because low-energy diets limit immune organ development [36,37].

Red meat is controversial because it is thought to lead to obesity and other diseases. However, red meat is rich in protein and trace elements, making it a good choice for developing countries [38]. The pH value of meat is an important factor affecting meat quality, and changes in pH affect the color and water retention [39]. In this study, the pH of the lamb carcasses decreased with time. This was mainly due to anaerobic respiration in the body, which promotes the conversion of glycogen to lactic acid [40]. However, the pH did not significantly differ between the three energy levels. Feed does not affect meat pH [41]. Meat quality can be judged intuitively through instrumental meat color, and meat color directly influences purchase intentions. Studies show that people pay more attention to a* than to L* when purchasing meat [42]. Instrumental meat color has been negatively correlated with feed energy levels [43]. In the current study, the meat color score of the Alpine Merino lambs decreased significantly as the energy level increased. Meat color is mainly determined by myoglobin, and higher myoglobin concentrations yield a darker muscle color [44]. When oxygen levels in the muscles drop, oxymyoglobin is converted to ferric myoglobin, and the muscle darkens [45].

Water loss rates can reflect the water-holding capacity of meat and are related to the tenderness and juiciness of the meat [46]. Dietary energy levels have little effect on meat’s water-holding capacity [47]. After death, protein degradation reduces the water flow in cells to the drip channel; therefore, meat with a high drip loss rate loses nutrients faster [48]. In this study, feed energy levels did not significantly affect the cooked meat rate, water loss, or drip loss. Thus, feed energy levels have little influence on nutrient loss and subsequent mutton processing [47]. Shear force is negatively correlated with tenderness [49] and increases with age [50], but is minimally affected by dietary treatment [51]. In this study, the shear force increased when the energy level increased, but not significantly, and the shear force was higher in the medium group than in the high group, likely owing to the greater body weights of the high group [52].

Red meat is rich in saturated fatty acids; thus, it is a poor choice for people with chronic diseases [53]. However, red meat is rich in unsaturated fatty acids, especially C18:1t9, which benefits heart health [54]. Fatty acids in meat vary greatly among different species as well as among the same species [10]. Gama et al. found that compared with those in San Yines sheep, the SFA concentrations of C12:0, C14:0, and C16:0 were lower in the longissimus thoracis muscle of Boer goats [55]. In addition to genetic influences, feeding methods can alter fatty acid distributions. Dietary energy levels affect muscle fiber types and, subsequently, intramuscular fat distribution [15,17,56]. After experimental sheep eat high-energy diets, the fatty acid and polyunsaturated fatty acid (PUFA) contents in the meat increase [57]. In this study, the C16:0, C18:0, and C18:1n9c contents were higher in Alpine Merino lambs, and C18:1 n9c was higher in the high group than in the other two groups. The SFA/UFA ratios were similar in the high and medium groups and lower than those in the low group. PUFAs are divided into n–3PUFAs and n–6PUFAs [52]; n–3PUFAs play important roles in preventing metabolic, inflammatory, and other diseases and cancers [58,59]. n–6PUFA is associated with a variety of diseases and promotes body fat accumulation [60,61]. Therefore, the n–6PUFA/n–3PUFA ratio is involved in meat quality scoring [62]. We found that the medium group had the highest PUFA/SFA ratio, and the n–6PUFA/n–3PUFA ratio in the mutton decreased as the dietary energy level increased. Appropriate energy levels can effectively reduce fat accumulation and increase the PUFA content in lambs [63]. Therefore, lamb meat from the high group would be a good choice for healthy people, and lamb meat from the medium group would be better for people with chronic diseases.

Red meat is rich in protein and contains various amino acids, which play important roles in human nutrition and health [64]. Flavor-enhancing amino acids are precursors of cooked meat flavor and are important for forming meat flavors [65]. Previous studies have shown that dietary energy levels do not affect amino acid contents in the muscles [43,47], and our study yielded similar results. No significant differences were found in flavor amino acid (FAA). We found that the EAA/TAA ratio of the longissimus dorsi muscles of the Alpine Merino lambs was ~46%, and the EAA/NEAA ratio exceeded 80%, which met the quality protein standard recommended by the Food and Agriculture Organization of the United Nations (FAO) [66,67].

## 5. Conclusions

We measured the growth performance, slaughter performance, organ weight, and meat quality of weaned male Alpine Merino lambs under different dietary energy levels. Compared with the low-energy diet, the high-energy diet improved the growth and slaughter performances of the lambs but reduced the muscle b* and a* scores. Lambs in the high group had the highest UFA contents in their muscles, and those in the medium group had the lowest SFA contents in their muscles. Therefore, meat from lambs fed the high-energy diet is suitable for healthy people, whereas meat from lambs fed the medium-energy diet is a better choice for people with chronic diseases.

## Figures and Tables

**Table 1:** Composition and nutrient level of experimental diets (air-dried basis).

Items	Content %		
	High	Medium	Low
Ingredients			
Corn stalks	20.00	20.00	20.00
Corn	35.00	35.00	35.00
Molasses	4.00	4.00	4.00
Cottonseed meal	2.00	4.50	7.00
Soybean meal	7.00	7.00	7.00
Extruded soybean	8.00	3.50	0.00
Rumen fat powder	1.50	0.75	0.00
Corn husk	9.00	13.25	15.00
Corn germ meal	10.50	9.00	9.00
Limestone	1.20	1.20	1.20
NaCl	0.70	0.70	0.70
Expanded urea	0.60	0.60	0.60
Premix	0.50	0.50	0.50
Total	100.00	100.00	100.00
Nutrient levels			
DM	90.39	89.46	90.23
CP	14.63	14.60	14.62
ME MJ/kg	10.50	10.10	9.70
Starch	24.33	24.05	24.32
NDF	31.80	40.42	51.27
ADF	17.46	23.85	29.74
Ash	10.17	8.67	8.23
P	0.27	0.28	0.28
Ca	0.71	0.61	0.52

Dietary trace element supplemental level (mg/kg): S 200; Fe 25; Zn 40; Cu 8; I 0.3; Mn 40; Se 0.2; Co 0.1. Dietary vitamin supplemental level (IU/kg): VA 940; VE 20. ME and starch were measured by calculation, and other components of the formula were obtained by laboratory. CP = crude protein; DM = dry matter; ME = metabolic energy; NDF = neutral detergent fiber; ADF = acid detergent fiber; Ash = crude ash.

**Table 2:** Lamb growth performance.

Items	High	Medium	Low	*p*-Value
Initial body weight kg	24.11 ± 0.28	24.03 ± 0.33	24.19 ± 0.23	0.986
20 d Body weight kg	26.31 ± 0.74	25.87 ± 0.89	26.12 ± 0.76	0.927
48 d Body weight kg	34.43 ± 0.81	33.85 ± 0.79	34.27 ± 0.83	0.873
76 d Body weight kg	43.17 ± 0.75	41.83 ± 0.75	40.84 ± 0.72	0.102
104 d Body weight kg	51.06 ± 0.60 ^a^	49.47 ± 0.73 ^ab^	48.46 ± 0.79 ^b^	0.048 *
20 d–48 d ADG g/d	324.64 ± 17.35	295.36 ± 19.48	284.29 ± 16.40	0.270
48 d–76 d ADG g/d	327.50 ± 27.11	269.64 ± 17.51	266.43 ± 26.04	0.147
76 d–104 d ADG g/d	282.76 ± 13.99	284.14 ± 17.14	273.45 ± 15.83	0.873

High = 10.5 MJ/kg; medium = 10.1 MJ/kg; low = 9.7 MJ/kg. ADG = average daily gain. Different lowercase letters on the same line mean a significant difference (*p* < 0.05); the same or no uppercase letters mean an insignificant difference (*p* ≥ 0.05). * *p* < 0.05.

**Table 3:** Slaughter performance of lambs.

Items	High	Medium	Low	*p*-Value
LWBS kg	50.73 ± 0.89 ^a^	49.01 ± 0.67 ^b^	48.16 ± 0.30 ^b^	0.044 *
Carcass weight kg	24.15 ± 0.40 ^a^	23.95 ± 0.43 ^a^	22.45 ± 0.31 ^b^	0.013 *
Dressing percentage %	47.57 ± 0.57 ^ab^	48.72 ± 0.61 ^a^	46.42 ± 0.40 ^b^	0.024 *

LWBS = live weight before slaughter. Different lowercase letters on the same line mean a significant difference (*p* < 0.05); the same or no uppercase letters mean an insignificant difference (*p* ≥ 0.05). * *p* < 0.05.

**Table 4:** Organ indexes of lambs.

Items	High	Medium	Low	*p*-Value
Heart g	226.94 ± 3.86	219.00 ± 5.62	214.31 ± 2.64	0.126
Liver g	898.40 ± 31.45	887.10 ± 16.33	851.85 ± 22.57	0.380
Spleen g	103.81 ± 3.38	103.19 ± 3.94	95.06 ± 2.58	0.144
Lung g	498.06 ± 8.26	502.25 ± 24.38	497.25 ± 7.99	0.971
Kidney g	154.31 ± 4.27	147.38 ± 3.45	144.88 ± 3.52	0.209
Left testis g	173.75 ± 8.27	183.44 ± 6.25	177.69 ± 10.29	0.720
Right testis g	181.88 ± 8.39	199.63 ± 6.62	195.38 ± 10.93	0.350
Testis g	355.63 ± 16.64	383.06 ± 12.72	373.06 ± 21.06	0.529
Rumen ^1^ kg	4.94 ± 0.15	4.54 ± 0.07	4.53 ± 0.20	0.115
Rumen ^2^ kg	1.29 ± 0.02	1.33 ± 0.03	1.33 ± 0.02	0.450
Duodenum cm	63.33 ± 2.40	62.33 ± 1.45	59.00 ± 3.61	0.348
Ileum cm	54.20 ± 1.36	54.60 ± 0.68	54.00 ± 1.18	0.928
Cecum cm	119.00 ± 9.13	114.00 ± 4.32	115.40 ± 5.80	0.845
Jejunum cm	29.89 ± 0.27	27.98 ± 1.56	28.78 ± 0.32	0.404
Colon cm	4.66 ± 013	4.63 ± 0.13	4.55 ± 0.22	0.888
Small intestine cm	148.95 ± 1.45	145.05 ± 1.48	143.06 ± 2.57	0.167
Large intestine cm	123.74 ± 9.26	118.55 ± 4.48	120.08 ± 3.92	0.841
Total length of intestine cm	267.65 ± 4.13	263.91 ± 7.38	259.96 ± 3.31	0.609

Rumen ^1^ = rumen with contents; rumen ^2^ = rumen without contents. Small intestine = duodenum + ileum + jejunum; large intestine = cecum + colon.

**Table 5:** Physical and chemical properties of lamb meat.

Items		High	Medium	Low	*p*-Value
45 min	L*	49.36 ± 0.35	49.62 ± 0.41	50.56 ± 0.31	0.067
	a*	20.12 ± 0.33 ^b^	20.51 ± 0.55 ^b^	21.97 ± 0.38 ^a^	0.016 *
	b*	16.58 ± 0.28	16.54 ± 0.23	17.24 ± 0.17	0.700
24 h	L*	49.38 ± 0.47	49.52 ± 0.56	50.98 ± 0.43	0.057
	a*	21.67 ± 0.58 ^b^	22.83 ± 0.74 ^ab^	24.21 ± 0.61 ^a^	0.032 *
	b*	20.01 ± 0.35 ^b^	20.91 ± 0.75 ^b^	22.73 ± 0.57 ^a^	0.010 *
pH	45 min	6.26 ± 0.03	6.26 ± 0.03	6.30 ± 0.02	0.427
	24 h	5.38 ± 0.04	5.35 ± 0.03	5.36 ± 0.02	0.648
Water losing rate %		24.75 ± 1.18	22.28 ± 0.97	24.26 ± 0.92	0.223
Drip loss %		1.24 ± 0.15	1.53 ± 0.20	1.34 ± 0.13	0.427
Cooked meat percentage %		58.30 ± 0.46	58.51 ± 0.33	58.40 ± 0.26	0.922
EMA cm^2^		17.99 ± 0.59	18.70 ± 0.46	16.75 ± 0.54	0.051
Backfat thickness mm		8.05 ± 0.59	7.87 ± 0.44	6.53 ± 0.34	0.074
Shear strength N		81.03 ± 2.80	90.36 ± 5.70	78.84 ± 3.19	0.133

L* = lightness; a* = redness; b* = yellowness. EMA = eye muscle area. Different lowercase letters on the same line mean a significant difference (*p* < 0.05); the same or no uppercase letters mean an insignificant difference (*p* ≥ 0.05). * *p* < 0.05.

**Table 6:** Fatty acid content in lamb muscle, mg/100 g.

Items	High	Medium	Low	*p*-Value
SFA	2006.09 ± 274.34	1524.27 ± 203.06	2196.57 ± 146.40	0.153
C4:0	1.67 ± 0.05	1.48 ± 0.09	1.38 ± 0.04	0.055
C10:0	4.88 ± 0.68	3.81 ± 0.56	5.35 ± 0.42	0.224
C12:0	3.77 ± 0.72	3.38 ± 0.72	4.26 ± 0.18	0.608
C13:0	0.81 ± 0.16	0.99 ± 0.09	0.63 ± 0.02	0.126
C14:0	93.20 ± 12.49	66.52 ± 22.27	87.79 ± 6.51	0.270
C15:0	11.00 ± 1.65	9.66 ± 1.98	13.14 ± 1.41	0.374
C16:0	1131.68 ± 152.75	846.50 ± 125.86	1183.89 ± 70.46	0.187
C17:0	43.98 ± 6.28	37.07 ± 7.38	45.18 ± 2.79	0.596
C18:0	602.36 ± 47.63 ^b^	545.63 ± 50.64 ^b^	837.38 ± 77.86 ^a^	0.031 *
C20:0	2.55 ± 0.24	2.26 ± 0.13	2.19 ± 0.34	0.589
C21:0	6.66 ± 0.39 ^b^	4.52 ± 0.44 ^b^	12.08 ± 1.07 ^a^	0.001 **
C22:0	1.31 ± 0.20	1.22 ± 0.05	1.38 ± 0.15	0.754
C23:0	1.60 ± 0.00	1.83 ± 0.63	2.00 ± 0.30	0.874
MUFA	1893.17 ± 179.15 ^a^	1475.72 ± 119.94 ^ab^	1274.39 ± 77.59 ^b^	0.030 *
C14:1	2.51 ± 0.29	2.05 ± 0.50	2.10 ± 0.09	0.593
C15:1	2.85 ± 0.12 ^b^	2.58 ± 0.05 ^b^	3.42 ± 0.15 ^a^	0.006 **
C16:1	67.18 ± 9.43	52.11 ± 10.96	53.11 ± 2.04	0.422
C17:1	33.00 ± 3.41	29.24 ± 3.80	26.69 ± 0.52	0.378
C18:1n9c	1616.79 ± 188.59	1247.04 ± 136.77	902.91 ± 85.91	0.063
C18:1n9t	155.42 ± 21.29	133.80 ± 29.39	258.92 ± 54.84	0.080
C20:1	3.41 ± 0.47 ^b^	5.47 ± 2.09 ^b^	31.31 ± 4.47 ^a^	0.000 **
C22:1n9	2.09 ± 0.26	1.88 ± 0.00	1.62 ± 0.18	0.513
C24:1	9.55 ± 0.46	8.66 ± 0.57	10.11 ± 0.57	0.232
PUFA	295.56 ± 11.62	284.47 ± 15.32	338.00 ± 37.98	0.335
C18:2n6c	3.00 ± 0.60 ^b^	2.52 ± 0.06 ^b^	5.07 ± 0.71 ^a^	0.019 *
C18:2n6t	206.48 ± 8.66	200.10 ± 15.38	246.95 ± 35.25	0.353
C18:3n6	3.76 ± 0.11	2.89 ± 0.24	4.55 ± 0.58	0.050
C18:3n3	10.76 ± 1.11	10.73 ± 2.53	15.16 ± 1.58	0.226
C20:2	3.40 ± 0.14 ^b^	3.31 ± 0.67 ^b^	7.47 ± 0.95 ^a^	0.007 **
C20:3	1.33 ± 0.09	1.25 ± 0.08	1.37 ± 0.15	0.719
C20:4n6	59.75 ± 2.53	58.32 ± 1.57	55.14 ± 2.75	0.416
C20:5n3	5.27 ± 0.13 ^a^	4.00 ± 0.30 ^b^	3.31 ± 0.16 ^b^	0.002 **
C22:6n3	2.56 ± 0.15	2.63 ± 0.05	2.44 ± 0.08	0.451
UFA	2194.74 ± 176.61 ^a^	1775.98 ± 86.87 ^b^	1629.25 ± 98.23 ^b^	0.022 *
SFA/UFA	0.92 ± 0.07 ^b^	0.91 ± 0.33 ^b^	1.33 ± 0.12 ^a^	0.039 *
PUFA/SFA	0.16 ± 0.02	0.20 ± 0.02	0.17 ± 0.02	0.313
n–6 PUFA	273.7 ± 11.28	264.34 ± 12.35	311.10 ± 35.42	0.336
n–3 PUFA	15.70 ± 0.64	14.46 ± 1.43	19.11 ± 1.33	0.074
Σn–6/Σn–3	16.89 ± 0.19	16.57 ± 1.30	17.28 ± 0.35	0.818

SFA = saturated fatty acid; UFA = unsaturated fatty acid; MUFA = monounsaturated fatty acid; PUFA = polyunsaturated fatty acid. 2n–6 PUFA = C18:2 c6 + C18:2 t6 + C18:3n–6 + C20:4n–6; n–3 PUFA = C18:3n–3 + C20:3n–3 + C20:5n–3+ C22:6n–3. Different lowercase letters on the same line mean a significant difference (*p* < 0.05); the same or no uppercase letters mean an insignificant difference (*p* ≥ 0.05). * *p* < 0.05; ** *p* < 0.01.

**Table 7:** Amino acid distribution in lamb muscle, %.

Items		High	Medium	Low	*p*-Value
Asp ^2^	Aspartic acid	1.56 ± 0.03	1.60 ± 0.03	1.58 ± 0.04	0.679
Thr ^1^	Threonine	0.70 ± 0.00	0.72 ± 0.02	0.72 ± 0.02	0.619
Ser ^2^	Serine	0.82 ± 0.02	0.80 ± 0.00	0.82 ± 0.02	0.619
Glu ^2^	Glutamic acid	3.10 ± 0.03	3.12 ± 0.06	3.18 ± 0.05	0.487
Gly ^2^	Glycine	0.82 ± 0.02	0.76 ± 0.03	0.80 ± 0.00	0.100
Ala ^2^	Alanine	1.00 ± 0.00	1.02 ± 0.02	1.02 ± 0.02	0.619
Cys ^2^	Cystine	0.20 ± 0.03	0.18 ± 0.02	0.16 ± 0.03	0.564
Val ^1,2^	Valine	1.28 ± 0.02	1.32 ± 0.02	1.30 ± 0.03	0.531
Met ^2^	Methionine	0.40 ± 0.00	0.42 ± 0.02	0.40 ± 0.00	0.397
Ile ^1,2^	Isoleucine	0.80 ± 0.00	0.86 ± 0.06	0.86 ± 0.06	0.088
Leu ^1,2^	Leucine	1.50 ± 0.03	1.52 ± 0.04	1.56 ± 0.03	0.420
Tyr ^1^	Tyrosine	0.50 ± 0.00	0.50 ± 0.00	0.52 ± 0.02	0.397
Phe ^1^	Phenylalanine	0.90 ± 0.00	0.92 ± 0.04	0.90 ± 0.00	0.756
His ^1^	Histidine	0.60 ± 0.00	0.64 ± 0.03	0.64 ± 0.03	0.300
Lys ^2^	Lysine	1.78 ± 0.02	1.84 ± 0.04	1.82 ± 0.05	0.546
Arg ^2^	Arginine	1.10 ± 0.00	1.08 ± 0.02	1.08 ± 0.02	0.619
Pro ^2^	Proline	0.70 ± 0.00	0.68 ± 0.02	0.70 ± 0.00	0.397
EAA		8.10 ± 0.00	8.23 ± 0.15	8.37 ± 0.19	0.435
FAA		13.28 ± 0.10	13.36 ± 0.19	13.46 ± 0.14	0.689
NEAA		9.70 ± 0.06	9.66 ± 0.12	9.74 ± 0.08	0.816
TAA		17.76 ± 0.12	17.98 ± 0.29	18.06 ± 0.21	0.608
EAA/TAA		45.34 ± 0.09	46.13 ± 0.16	46.13 ± 0.36	0.083
EAA/NEAA		82.94 ± 0.28	85.65 ± 0.54	85.63 ± 1.24	0.085

The number superscript ^1^ means EAA, essential amino acid; the number superscript ^2^ means FAA, flavor amino acid; TAA, total amino acid; NEAA, nonessential amino acid = TAA−EAA.

## Data Availability

The authors confirm that the data supporting the findings of this study are available within the article.
